# Noise reduction in brain magnetic resonance imaging using adaptive wavelet thresholding based on linear prediction factor

**DOI:** 10.3389/fnins.2024.1516514

**Published:** 2025-01-10

**Authors:** Ananias Pereira Neto, Fabrício J. B. Barros

**Affiliations:** ^1^Federal Institute of Education, Science and Technology of Pará - IFPA, Belém, Brazil; ^2^Graduate Program in Electrical Engineering, Federal University of Pará - UFPA, Belém, Brazil

**Keywords:** wavelet transform, wavelet thresholding, image noise reduction, adaptive thresholding, MSE, PSNR, SSIM

## Abstract

**Introduction:**

Wavelet thresholding techniques are crucial in mitigating noise in data communication and storage systems. In image processing, particularly in medical imaging like MRI, noise reduction is vital for improving visual quality and accurate analysis. While existing methods offer noise reduction, they often suffer from limitations like edge and texture loss, poor smoothness, and the need for manual parameter tuning.

**Methods:**

This study introduces a novel adaptive wavelet thresholding technique for noise reduction in brain MRI. The proposed method utilizes a linear prediction factor to adjust the threshold adaptively. This factor leverages temporal information and features from both the original and noisy images to determine a weighted threshold. This dynamic thresholding approach aims to selectively reduce or eliminate noise coefficients while preserving essential image features.

**Results:**

The proposed method was rigorously evaluated against existing state-of-the-art noise reduction techniques. Experimental results demonstrate significant improvements in key performance metrics, including mean squared error (MSE), peak signal-to-noise ratio (PSNR), and structural similarity index (SSIM).

**Discussion:**

The proposed adaptive thresholding technique effectively addresses the limitations of existing methods by providing a more efficient and accurate noise reduction approach. By dynamically adjusting the threshold based on image-specific characteristics, this method effectively preserves image details while effectively suppressing noise. These findings highlight the potential of the proposed method for enhancing the quality and interpretability of brain MRI images.

## 1 Introduction

Medical imaging plays a critical role in modern diagnostics, particularly magnetic resonance imaging (MRI), which offers high-resolution and detailed imaging of the human body (Chang et al., 2024). However, the noise introduced during the image acquisition process can make it difficult for the specialist to accurately analyze and interpret these images, potentially compromising the quality of the diagnosis. In this context, noise reduction in MRI becomes essential for enhancing the accuracy and reliability of the diagnostic outcomes (Mishro et al., 2022). One promising approach for reducing noise in medical images is through the use of adaptive wavelets, which are powerful tools in signal and image processing, particularly effective for MRI noise reduction (Zhang et al., 2019; Golilarz et al., 2020; Assam et al., 2021; Juneja et al., 2021; Sonia and Sumathi, 2022; Benhassine et al., 2021).

To maximize the advantages of the wavelet transform and mitigate noise effects, adaptive wavelet thresholding methods rely on several parameters, including the standard deviation of the noise, length of the processed signal, and decomposition level of the wavelet transform, which estimate the signal's properties in the frequency domain (Sahoo et al., 2024). However, current adaptive wavelet thresholding methods often fail to incorporate readily available temporal information from the signal sequence. This omission can lead to issues, especially when processing signals with high motion intensity because these methods primarily target signals with low to moderate motion intensity. Consequently, current wavelet thresholding techniques lack parameters to exploit the correlation between noisy and non-noisy coefficients, thus making them less effective for noise elimination in the time domain.

The original wavelet thresholding method for noise reduction was developed by Donoho and Johnstone (Donoho and Johnstone, 1994; Donoho, 1995; Donoho and Johnstone, 1995). Their research produced several thresholding methods, the most notable being the universal threshold, which provides an optimal estimate to minimize errors in wavelet coefficient thresholding during signal processing. This approach, also known as the VisuShrink threshold estimator, is characterized by its simple and efficient implementation. Building on the foundational work of Donoho and his collaborators, Chang et al. (2000) proposed a threshold derived from a Bayesian framework known as the BayesShrink. The BayesShrink threshold is simple and adaptable to each wavelet sub-band using data-based estimates, selecting parameters based on the minimum description length criterion. This approach significantly aids in noise reduction by employing a smooth wavelet threshold. Many studies have shown the importance of wavelet-based methods for noise reduction, particularly in brain MRI. These studies focus on optimizing the choice of wavelets for noise reduction, as well as the development of new threshold functions and values to improve performance.

Sun et al. (2019) proposed a novel threshold function for the wavelet transform based on the Gaussian Kernel function combined with the soft threshold function. This approach utilizes a squared exponential kernel, also known as radial basis function or Gaussian kernel, providing enhanced flexibility and adaptability over other thresholding techniques. The proposed function establishes optimal threshold conditions that preserve image details while minimizing noise. It offers significant advantages over traditional methods like smooth thresholding by addressing common issues such as discontinuity, edge loss, and inadequate smoothness during image processing.

Zhang et al. (2019) proposed a noise reduction method for brain MRI using a new threshold function that combines the advantages of soft and hard thresholding, with adjustments made through the parameter *k*. Their methods avoid the oscillation phenomenon often introduced by noise reduction. By carefully tuning α, the accuracy of threshold estimation improves: when α is low, a high-frequency threshold is calculated, resulting in a slightly higher threshold, whereas a high α yields a lower frequency threshold.

Golilarz et al. (2019) utilized a nature-inspired Harris Hawks Optimization (HHO) algorithm to optimize the parameters of the threshold function. This optimization allows for the refinement of wavelet coefficients before applying the inverse wavelet transform. In addition, the authors introduced a threshold based on the adaptive generalized Gaussian distribution (AGGD), a data-driven function with an adaptive threshold value used to improve image quality during the noise reduction process. The AGGD threshold function, guided by a non-linear smooth function, enhances image quality by ensuring that the function remains fully non-linear and distinct from previous approaches. Golilarz et al. (2020) further refined their work with a wavelet thresholding method based on the enhanced AGGD from Golilarz et al. (2019) and specifically designed for noise elimination in brain MRI. The enhanced AGGD thresholding function focuses on Gaussian distribution, providing a flexible, non-linear, data-based approach suitable for any image type. Instead of setting coefficients to zero within the interval [-T, T], the function adjusts non-relevant coefficients using the AGGD threshold function, leading to improved noise reduction.

A state-of-the-art review of digital image processing techniques, with an emphasis on artifact processing and reconstruction approaches, some recent research has been proposed, such as Yin and Chen (2024), has developed a noise reduction network for hyperspectral images (HSI) based on a new deep unfolding network inspired by CD-CSC (Content-Dependent 3-D Convolutional Sparse Coding) called CD-CSCNet. Deep unfolding is a viable way of improving the interpretability of the deep network. In the proposal developed, the method represents the spatial-spectral ensemble for reducing HSI noise, mitigating the unpleasant effect of spectral distortion. Extensive experimental results on the datasets demonstrate that CD-CSCNet outperforms several recent pure data-driven and DU-based networks quantitatively and visually. Rong et al. (2024) proposes a two-stage super-resolution image reconstruction network comprising encoding and decoding stages. To mitigate the impact of these reconstruction artifacts, the study introduces the Residual Dense Feature Aggregation Network (SR-RDFAN-LOG), specifically designed to enhance the resolution of registration images. By integrating deep and shallow features, the proposed method improves image resolution and leverages implicit neural representation to generate registration images at arbitrary resolutions. Consequently, the method outperforms existing techniques in terms of PSNR, SSIM, and LPIPS metrics for registration image resolution enhancement. El-Shafai et al. (2024) analyzes various noise reduction methods for medical images, comparing traditional and deep learning (DL) techniques. The study presents a comparative analysis to highlight the strengths and weaknesses of each method. Experimental results demonstrate that DL methods, particularly those utilizing convolutional neural networks (CNNs), achieve optimal performance in noise reduction for medical images.

In noise reduction methods, selecting appropriate thresholds and developing new threshold functions are important features. In this work, a new threshold and threshold function for noise reduction in brain MRI, are proposed, incorporating additional parameters that account for wavelet decomposition levels and especially, the addition of the linear prediction factor. This factor represents a weighted estimate of the threshold minimizing the prediction error derived from the time difference between the noisy and original images. The method calculates a linear prediction factor to reduce the average mean squared error (MSE) between the noisy and original images. The key contributions of this study are as follows: (1) A new threshold estimator and wavelet threshold function are developed to improve brain MRI noise reduction by leveraging the temporal information and characteristics of the noisy and original images to reduce noise in the temporal domain. (2) The threshold proposed in this work extends the universal threshold, incorporating two innovative parameters: the decomposition level *j* of the wavelet transform and the linear prediction factor β. The *j* parameter controls the depth of the multiresolution analysis, making it possible to adjust the separation between the low and high-frequency components of the image. The β factor, in turn, introduces a linear prediction mechanism that aims to minimize the mean squared error (MSE) between the original and noise image, adapting the threshold more precisely to the specific characteristics of the image. (3) The new threshold estimator adapts to the different sub-band characteristics of the wavelet transform for each image, adjusting the wavelet coefficients based on the noisy image conditions, considering the weighting of the linear prediction factor and the level decomposition. (4) The proposed adaptive wavelet thresholding method incorporates the correlation between the noisy and original images to calculate a cost function that minimizes the prediction error. This linear prediction factor improves the accuracy of wavelet coefficient selection near the threshold. 5. The new adaptive threshold function avoids the limitations of the hard threshold (discontinuity), and the soft threshold (loss of original signal characteristics and edges blurring of image). It also minimizes oscillation effects, such as the pseudo-Gibbs phenomenon, often associated with hard and soft thresholds.

## 2 Materials and methods

### 2.1 Noise reduction with wavelet threshold

The wavelet transform has prompted numerous applications across various disciplines, predominantly due to its fundamental wavelet functions and properties such as energy compaction and localization in the time frequency domain. These properties are highly beneficial in signal processing applications, including voice, radar signals, images, and video (Shanthamallappa et al., 2024; Taranenko and Oliinyk, 2024; Dizon and Hogan, 2024; Zhou et al., 2023; Chen, 2024; Zhou et al., 2020; Li et al., 2024; Huang and Dragotti, 2022; Li et al., 2022; Chen and Krzyzak, 2024). The wavelet thresholding technique involves adjusting wavelet coefficients to reduce or eliminate unwanted noise or interference in communication systems, including computer applications and digital storage. During signal processing, various forms of interference can alter the information, and wavelet thresholding helps mitigate these effects.

Wavelet thresholding is a simple technique with a wide range of customizable options and parameters that can be adjusted to reduce the probability of processing errors. Therefore, coefficients that are irrelevant to the processed signal, often determined by a threshold, are either reduced or nullified (Bnou et al., 2020). Different thresholding methods have been proposed in the literature, as the choice of threshold significantly impacts noise reduction and the preservation of the signal's visual characteristics. Donoho (1995) proposed using of orthonormal wavelet basis functions to remove signal noise, a procedure they called denoising. Their denoising procedure takes advantage of the wavelet transform's energy concentration property, where the signal's useful information is represented by a small number of coefficients, whereas noise is distributed across many of them. In the wavelet domain, the noise-contaminated coefficients possess variances equal to the noise present in time-domain signal samples. However, the signal energy is confined to only a few coefficients. As a result, the noise level can be reduced by zeroing out the expansion coefficients that fall below a specific threshold, directly influencing the effectiveness of the denoising method. According to Donoho and Johnstone (1994), when the threshold parameter is selected correctly, the method can produce excellent results across various types of signals. In addition, the choice of wavelet family is critical when applying the wavelet transform. Several families of wavelet functions can be used for decomposition, as long as they meet specific criteria. The objective is to ensure that the selected wavelet family concentrates the energy of the helpful signal into a few coefficients of expansion. In this case, the higher the concentration, the more coefficients can be eliminated during processing without causing significant distortion, thus leading to effective noise reduction.

**Figure 1:**
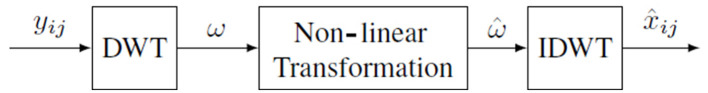
Block diagram of the wavelet threshold noise reduction method.

In the denoising process, the primary challenge is estimating the original signal, such as an image {*x*_*ij*_, *i, j* = 1,..., *N*}, from its noisy observations {*y*_*ij*_, *i, j* = 1,..., *N*}, expressed by Equation 1:


(1) $$\begin{array}{l} y_{ij}=x_{ij}+\varepsilon_{ij} \end{array}$$

where {ε_*ij*_, *i, j* = 1,..., *N*} represents the noise modeled by a stationary Gaussian stochastic process with zero mean and variance σ^2^, *N*(0, σ^2^) (Al-azzawi, 2020; Yilmaz, 2020). The wavelet denoising method involves applying the discrete wavelet transform (DWT) to the noisy signal *y*_*ij*_ and processing the resulting transform coefficients through a nonlinear filter. The amplitudes of these coefficients are compared against a threshold *T*. The estimate of the original signal *x*_*ij*_, denoted by ${\hat{x}}_{ij}$, is obtained by performing the inverse DWT of the threshold coefficients, as illustrated by the block diagram in Figure 1.

In this diagram, ω represents the vector containing the DWT coefficients of *y*, given by Equation 2:


(2) $$\begin{array}{l} \omega =Wy \end{array}$$

where *W* is the two-dimensional linear operator responsible for performing the dyadic orthogonal wavelet transformation. Similarly, applying the operator *W* em *x* and ε yields the wavelet coefficients *Wx* for the noiseless signal and *W*ε of the noise, respectively. Because denoising procedures use orthonormal wavelet basis functions, we have the following relationship (Equation 3):


(3) $$\begin{array}{l} W^{-1}W=I \end{array}$$

where *W*^−1^ is the inverse wavelet transform operator, and *I* is the identity matrix. The denoising method filters each coefficient *y*_*ij*_ in the detail sub-bands using a threshold function to obtain *x*_*ij*_. Thus, the estimated signal $\hat{x}$ in the wavelet denoising method is given by Equation 4:


(4) $$\begin{array}{l} \hat{x}=W^{-1}\hat{\omega } \end{array}$$

According to Donoho and Johnstone (1994), two procedures are proposed to modify the wavelet coefficients based on their comparison with threshold *T*, denoted as ${\hat{\omega }}_{ij}$. Therefore, the wavelet coefficients ${\hat{\omega }}_{ij}$ are processed by the threshold functions, with one approach using a hard threshold and the other using a soft threshold. In the hard threshold method, coefficients greater than or equal to the threshold are retained, while coefficients below the threshold are discarded, as defined by Equation 5:


(5) $$\begin{array}{l} {\hat{\omega }}_{ij}=\{\begin{array}{cc} \omega_{ij},\; & |\omega_{ij}|\geq T\\ 0,\; & |\omega_{ij}|<T \end{array} \end{array}$$

In the soft threshold method, if a coefficient's amplitude is smaller than the threshold, it is set to zero. Otherwise, its amplitude is reduced by an amount equal to the threshold, as defined by Equation 6:


(6) $$\begin{array}{l} {\hat{\omega }}_{ij}=\{\begin{array}{cc} sgn\left(\omega_{ij}\right)\left(|\omega_{ij}|-T\right),\; & |\omega_{ij}|\geq T\\ 0,\; & |\omega_{ij}|<T \end{array} \end{array}$$

where *sgn(.)* denotes the sign function.

A third thresholding method introduces the parameter α, which is between 0 and 1. This approach, called semi-soft thresholding (Wang et al., 2021), calculates wavelet coefficients based on a combination of the hard and soft thresholding methods, as described (Equation 7):


(7) $$\begin{array}{l} {\hat{\omega }}_{ij}=\{\begin{array}{cc} sgn\left(\omega_{ij}\right)\left(|\omega_{ij}|-\alpha T\right),\; & |\omega_{ij}|\geq T\\ 0,\; & |\omega_{ij}|<T \end{array} \end{array}$$

The performance of wavelet-based denoising techniques depends chiefly on the threshold *T* and strategy for adjusting the coefficients. Donoho and Johnstone (1994) and Donoho (1995), proposed the universal threshold, which, according to the authors, has been shown to deliver good performance across a range of signals. The universal threshold is defined by Equation 8:


(8) $$\begin{array}{l} T=\sigma \sqrt{2logN} \end{array}$$

where *N* is the length of the input signal and σ is the estimated noise level. This noise level is typically estimated from the wavelet coefficient data, as well as a robust estimator for σ, based on the median of the wavelet coefficients at the finest decomposition level (Donoho and Johnstone, 1994; Donoho, 1995), as expressed by Equation 9:


(9) $$\begin{array}{l} \sigma =\frac{median\left(\left|\omega_{ij}\right|\right)}{0.6745} \end{array}$$

where ω_*ij*_ is the *HH*_1_. *HH*_1_ represents the diagonal detail coefficients at the first level of wavelet decomposition. The universal threshold is designed to minimize the error associated with the wavelet coefficient thresholding process. This method is known as the VisuShrink threshold estimator, as described (Equation 8).

The work of Donoho and Johnstone (1994) has led to the development of numerous denoising methods, with SureShrink and BayesShrink being primary examples. The SureShrink method was developed by Donoho and Johnstone (1995) and is based on the Stein (1981)'s unbiased risk estimator (SURE), which determines a sub-band adaptive threshold for wavelet coefficients that minimizes noise while preserving signal details. Meanwhile, BayesShrink (Chang et al., 2000), developed later, builds on an adaptive thresholding approach, such as SureShrink, but with a Bayesian framework. It assumes that the wavelet coefficients follow a generalized Gaussian distribution. The threshold in the BayesShrink method is derived by minimizing the Bayesian risk, balancing the trade-off between denoising and retaining image details. Compared to SureShrink, BayesShrink performs better in minimizing the MSE by applying a threshold that adapts to each individual sub-band at a given image resolution level. BayesShrink's threshold is derived from risk minimization, and is given by Equation 10:


(10) $$\begin{array}{l} T=\frac{{\hat{\sigma }}^{2}}{\hat{\sigma_{x}}} \end{array}$$

where ${\hat{\sigma }}^{2}$ is the variance of the estimated noise level for the *HH*_1_ sub-band, calculated using Equation 9, and $\hat{\sigma_{x}}$ is the standard deviation of the wavelet coefficients, which is calculated using Equation 11:


(11) $$\begin{array}{l} \hat{\sigma_{x}}=\sqrt{max\left({\hat{\sigma_{y}}}^{2}-{\hat{\sigma }}^{2},0\right)} \end{array}$$

where ${\hat{\sigma_{y}}}^{2}$ is the variance of the wavelet coefficients ω_*ij*_ determined by means of Equation 12:


(12) $$\begin{array}{l} {\hat{\sigma_{y}}}^{2}=\frac{1}{N^{2}}\overset{K}{\underset{i,j=1}{\sum }}\omega_{ij}^{2} \end{array}$$

where *N* represents the number of wavelet coefficients in the sub-band, and ω_*ij*_ are the individual wavelet coefficients.

BayesShrink is considered one of the most efficient denoising methods for the processing of two-dimensional signals, such as images and videos because it provides more efficient noise reduction and retains more image detail. Given its performance and the simplicity of its implementation, BayesShrink is widely regarded as one of the most effective methods for reconstructing 2D signals (Chang et al., 2000).

### 2.2 Proposed threshold estimator

The threshold proposed in this work builds upon the universal threshold by adding two additional parameters: the scale or decomposition level of the wavelet transform and the linear prediction factor, β. The linear prediction factor is included to minimize the MSE between the noisy and original images. This new threshold, when applied to the thresholding function, adjusts the wavelet coefficients more effectively to reduce noise. The proposed threshold is expressed in Equation 13:


(13) $$\begin{array}{l} T=\frac{\sigma \sqrt{2logN}}{log{\left(1+j\right)}^{\beta }} \end{array}$$

where σ is the noise standard deviation, *N* is the signal length, *j* is the decomposition scale, and β is the linear prediction factor determined by the prediction error, which represents the temporal difference between the noisy image *I*_*n*_*[m, n*, *t*_2_*]* and the original image *I*_*o*_*[m, n*, *t*_1_*]*. The incorporation of the linear prediction factor β, together with the decomposition level, enhances the efficiency of the threshold *T*, resulting in more effective noise reduction at different wavelet transform scales.

The threshold *T* is a central parameter in the wavelet thresholding technique for noise reduction. It acts as a cut-off value that distinguishes the relevant wavelet coefficients from those attributed to noise. The effectiveness of this approach depends on how the threshold is determined and applied when filtering the coefficients. The main aim of estimating the threshold T is to eliminate the wavelet coefficients considered irrelevant to the signal representation, usually coefficients with minimum values that are noise. This threshold parameter can be adjusted according to various factors, which directly affect the quality of the noise reduction and the preservation of the original signal's characteristics. In the present proposal, the main factors are the linear prediction factor and the wavelet transform decomposition level.

### 2.3 Linear prediction factor

The threshold estimator described in Equation 13 was developed to estimate wavelet coefficients, as well as to adapt to varying sub-band characteristics based on the conditions of both the noisy and original images. The linear prediction factor, β, is calculated by minimizing the MSE between the noisy and original images. In this context, the prediction error represents the time difference between the noisy image *I*_*n*_*[m, n*, *t*_2_*]* and the original image *I*_*o*_*[m, n*, *t*_1_*]*, weighted by the factor β. The prediction error is given by Equation 14:


(14) $$\begin{array}{l} e_{prediction}\left[m,n\right]=I_{n}\left[m,n,t_{2}\right]-\beta I_{o}\left[m,n,t_{1}\right] \end{array}$$

The linear prediction factor β is obtained by minimizing the MSE between the noisy and original images, as expressed in Equation 15:


(15) $$\begin{array}{l} \beta =\min E\left\{e_{prediction}^{2}\left[m,n\right]\right\} \end{array}$$

By solving the differential equation with respect to β in Equation 16 and setting the derivative equal to zero in Equation 17, we obtain the linear prediction factor β:


(16) $$\begin{array}{l} \frac{\partial E\left\{e_{prediction}^{2}\right\}}{\partial \beta }={\frac{\partial E\left\{I_{n}\left[m,n,t_{2}\right]-\beta I_{o}\left[m,n,t_{1}\right]\right\}}{\partial \beta }}^{2} \end{array}$$

(17) $$\begin{array}{l} 2E\left\{\left[\left(I_{n}\left[m,n,t_{2}\right]-\beta I_{o}\left[m,n,t_{1}\right]\right)\right]\left(-I_{o}\left[m,n,t_{1}\right]\right)\right\}=0 \end{array}$$

Thus, the β factor that minimizes the MSE between the noisy and original images is given by Equation 18:


(18) $$\begin{array}{l} \beta^{*}=\frac{E\left\{I_{o}\left[m,n,t_{1}\right]I_{n}\left[m,n,t_{2}\right]\right\}}{E\left\{{I_{o}}^{2}\left[m,n,t_{1}\right]\right\}} \end{array}$$

Assuming that both the noisy and original images have non-zero random means and their joint probability distribution is unknown, the linear prediction factor β is given by Equation 19:


(19) $$\begin{array}{l} \beta =\frac{\sum_{m=1}^{M}\sum_{n=1}^{N}I_{o}\left[m,n,t_{1}\right]I_{n}\left[m,n,t_{2}\right]}{\sum_{m=1}^{M}\sum_{n=1}^{N}{I_{o}}^{2}\left[m,n,t_{1}\right]} \end{array}$$

### 2.4 New adaptive thresholding function

In the classical thresholding methods proposed by Donoho, both the hard and soft threshold functions exhibit certain limitations. The hard threshold function preserves the critical information from the original signal, but it introduces discontinuities in the noise wavelet coefficients, causing visual distortions in images due to the pseudo-Gibbs phenomenon. Conversely, the soft threshold function, unlike the hard threshold, ensures continuity by keeping the noise wavelet coefficients proportional to the image coefficients, thereby reducing the pseudo-Gibbs effect. However, when using soft thresholding for noise reduction, the reconstructed signal often loses significant features, such as sharp edges, resulting in blurred details. This blurring occurs because the substantial noise wavelet coefficients are overly attenuated when processed against the threshold. In conventional thresholding methods, selecting an optimal threshold is difficult. If the threshold is set too high, significant image details are lost; if it is too low, residual noise remains. To overcome these challenges and improve noise reduction while preserving crucial image details and minimizing the pseudo-Gibbs phenomenon, this work proposes a new adaptive thresholding function, as defined in Equation 20:


(20) $$\begin{array}{l} {\hat{\omega }}_{ij}=\{\begin{array}{cc} sgn\left(\omega_{ij}\right)\left(|\omega_{ij}|-\sin\left(\frac{\pi }{2}{\left(\frac{T}{\left|\omega_{ij}\right|}\right)}^{\frac{\beta }{T}}\right)T\right), & |\omega_{ij}|\geq T\\ 0, & |\omega_{ij}|<T \end{array} \end{array}$$

where ω_*ij*_ and *T* represent the wavelet coefficients of the noisy image and threshold, respectively, and ${\hat{\omega }}_{ij}$ is the resulting coefficient after processing with the new adaptive threshold function. The term *sgn(*ω_*ij*_*)* denotes the sign of ω_*ij*_, whereas β is the linear prediction factor.

**Figure 2:**
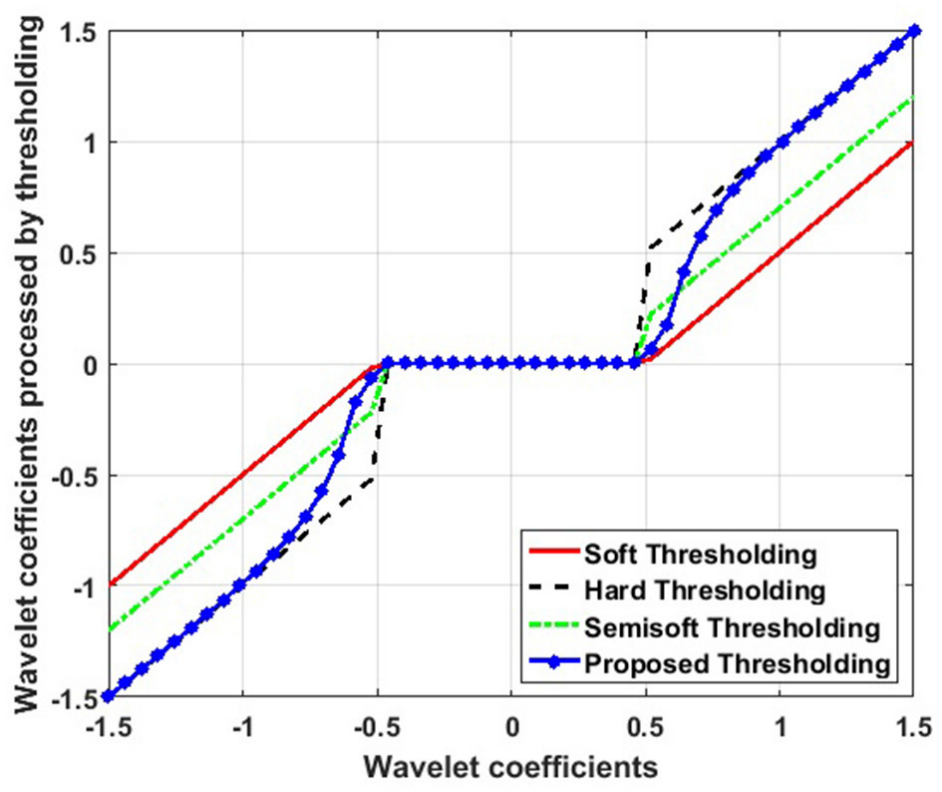
Comparison between hard, soft, semisoft, and the newly proposed threshold functions.

Figure 2 illustrates the differences between hard, soft, semisoft, and newly proposed threshold functions. As can be observed, the hard threshold function shows discontinuity at the threshold *T*, causing oscillations in the noisy image reconstruction. Unlike the hard threshold, the soft threshold function avoids discontinuity. However, the resulting reconstructed image may deviate from the original owing to the excessive attenuation of the noise wavelet coefficients.

In the proposed new threshold function, as |ω_*ij*_| → *T*, the value of ${\hat{\omega }}_{ij}$ progressively approaches zero, that is, ${\hat{\omega }}_{ij}$→*0*. Therefore, the new threshold function maintains continuity at the threshold *T*, helping to reduce the Gibbs phenomenon. Similarly, as |ω_*ij*_| → ∞, ${\hat{\omega }}_{ij}$ converges to ω_*ij*_, retaining the properties of the hard threshold function. Consequently, the difference between ${\hat{\omega }}_{ij}$ and ω_*ij*_ decreases significantly, minimizing the constant bias effect observed in the soft threshold function.

The value of the β factor is adjusted based on the linear prediction between the noisy and original images, allowing the new threshold function to better adapt to the specific characteristics of each image. As a result, the new threshold function overcomes the limitations of both soft and hard thresholds, providing a continuous and smooth function that effectively reduces Gibbs oscillations and mitigates noise effects in the image.

## 3 Results

**Figure 3:**
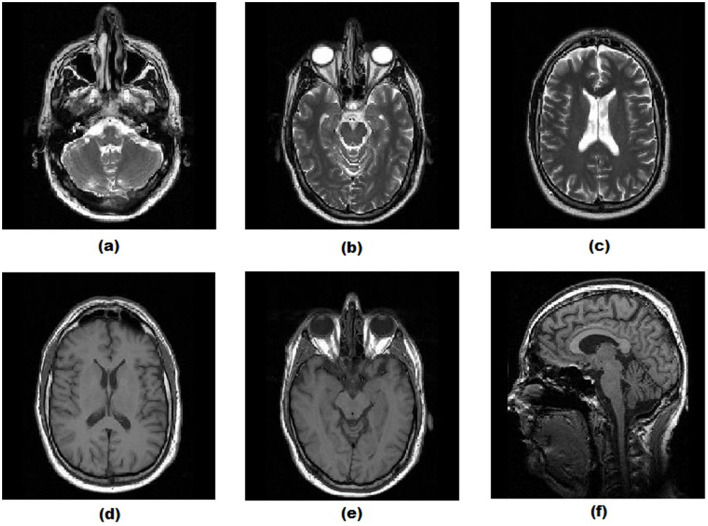
Original brain magnetic resonance images: **(A)** MRI Brain Image 1, **(B)** MRI Brain Image 2, **(C)** MRI Brain Image 3, **(D)** MRI Brain Image 4, **(E)** MRI Brain Image 5, and **(F)** MRI Brain Image 6.

To evaluate the efficiency of the proposed adaptive thresholding method, it was benchmarked against several established noise reduction methods using six MRI brain images from the ethical magnetic resonance brain images (Dataset, 2024; Figure 3). The comparison involved introducing Gaussian white noise with additive variance ranging from 0.01 to 0.05. The proposed method was evaluated alongside *VisuShrink* (Donoho and Johnstone, 1994), *BayesShrink* (Chang et al., 2000), Gaussian Kernel (Sun et al., 2019), Improved Threshold (Zhang et al., 2019), and AGGD (Golilarz et al., 2020) utilizing the biorthogonal wavelet base *bior3.9* with three decomposition levels. All implementations and experiments were carried out in MATLAB R2020a.

### 3.1 Quantitative assessment

The quantitative assessment employed three objective metrics: (1) MSE, which represents the average squared difference between the original and reconstructed images and measures error severity; (2) peak signal-to-noise ratio (PSNR), which measures the quality of the reconstructed image about the original image (Chan and Whiteman, 1983; Ferreira et al., 2024); and (3) structural similarity index (SSIM), which is used to measure the similarity between the original and processed images by analyzing important visual properties such as brightness, contrast, and structure (Wang et al., 2004; Bhatt et al., 2021). SSIM is considered a more comprehensive method than PSNR and MSE, as it accounts for essential characteristics of the human visual system (HSV). PSNR is calculated as expressed in Equation 21 for an original image *x* and a reconstructed image *y* of dimension *M x N*.


(21) $$\begin{array}{l} \text{PSNR (x,y)}=10\log_{10}\left[\frac{L^{2}}{MSE}\right] \end{array}$$

where *L* is the dynamic range of the image (e.g., *L* = 255 for an 8-bit image with 256 gray levels). MSE is defined in Equation 22:


(22) $$\begin{array}{l} \text{MSE}=\frac{1}{MxN}\overset{M}{\underset{m=1}{\sum }}\overset{N}{\underset{n=1}{\sum }}|x_{\left(m,n\right)}-y_{\left(m,n\right)}{|}^{2} \end{array}$$

SSIM, which measures the similarity between the original image *x* and reconstructed image *y*, can be calculated as in Equation 23:


(23) $$\begin{array}{l} \text{SSIM (x,y)}=\frac{\left(2\mu_{x}\mu_{y}+C_{1}\right)\left(2\sigma_{xy}+C_{2}\right)}{\left(\mu_{x}^{2}+\mu_{y}^{2}+C_{1}\right)\left(\sigma_{x}^{2}+\sigma_{y}^{2}+C_{2}\right)} \end{array}$$

where μ_*x*_ and μ_*y*_ represent the means of *x* and *y*, $\sigma_{x}^{2}$ and $\sigma_{y}^{2}$ are their variances, and σ_*xy*_ is the covariance of *x* and *y*. *C*_1_ and *C*_2_ x'are constants used to stabilize the expression.

Table 1 presents the average MSE, PSNR, and SSIM values obtained from the different noise reduction wavelet methods for MRI Brain Image 1 and MRI Brain Image 2. According to the results, the proposed noise reduction technique outperformed other wavelet-based methods. By incorporating the linear prediction factor β in the threshold estimator, the method efficiently reduces image noise, yielding superior MSE, PSNR, and SSIM values compared to those obtained with the other methods.

**Table 1:** MSE, PSNR(dB), and SSIM results for all wavelet methods; MRI Brain Images 1 and 2.

**Image**	***MRI Brain Image 1***					***MRI Brain Image 2***				
Noise variance (σ)	0.01	0.02	0.03	0.04	0.05	0.01	0.02	0.03	0.04	0.05
**MSE**										
*Proposed*	**29**	**31**	**32**	**33**	**34**	**27**	**31**	**33**	**33**	**34**
*VisuShrink*	259	415	571	726	868	235	391	543	692	839
*BayesShrink*	89	116	129	139	145	88	115	129	138	144
*Gaussian Kernel*	90	115	129	137	143	89	114	127	136	143
*Improved Threshold*	91	116	129	138	144	90	115	129	137	143
*AGGD*	32	35	36	37	38	32	35	37	38	39
**PSNR(dB)**										
*Proposed*	**33.55**	**33.12**	**33.01**	**32.93**	**32.86**	**33.74**	**33.21**	**33.00**	**32.97**	**32.88**
*VisuShrink*	23.99	21.95	20.56	19.52	18.75	24.43	22.20	20.78	19.73	18.89
*BayesShrink*	28.62	27.49	27.01	26.71	26.50	28.71	27.54	27.03	26.75	26.54
*Gaussian Kernel*	28.59	27.53	27.03	26.77	26.57	28.65	27.55	27.08	26.79	26.59
*Improved Threshold*	28.53	27.47	27.01	26.73	26.55	28.61	27.52	27.03	26.77	26.59
*AGGD*	33.09	32.72	32.57	32.47	32.39	33.10	32.65	32.48	32.33	32.26
**SSIM**										
*Proposed*	**0.520**	**0.439**	**0.393**	**0.363**	**0.342**	**0.502**	**0.421**	**0.373**	**0.346**	**0.317**
*VisuShrink*	0.476	0.390	0.339	0.306	0.284	0.451	0.363	0.317	0.285	0.263
*BayesShrink*	0.475	0.394	0.347	0.318	0.295	0.451	0.367	0.322	0.294	0.271
*Gaussian Kernel*	0.469	0.387	0.341	0.312	0.290	0.444	0.361	0.315	0.287	0.264
*Improved Threshold*	0.467	0.385	0.341	0.312	0.288	0.444	0.360	0.316	0.287	0.264
AGGD	0.444	0.366	0.321	0.291	0.271	0.416	0.337	0.296	0.267	0.244

The values in bold show better results compared to the other methods.

As observed in Table 1, for MRI Brain Image 1, the MSE value was approximately 32, corresponding to noise variances between 0.01 and 0.05. The proposed method demonstrated better performance, with a difference of less than 11% in the MSE relative to the second-best method, the AGGD, which presented an average MSE of 36. Regarding the PSNR, the proposed technique achieved an average of 33.10 dB, the highest among all methods tested. For SSIM, the proposed method also showed superior visual quality, with an average score of 0.411, indicating a significantly better image quality, contrasted with other techniques. Therefore, the method based on the linear prediction factor β effectively preserves the structure of the processed image while reducing noise.

Based on the analysis of the MSE results presented in Table 1, for MRI Brain Image 2, it was observed that the proposed method is quite effective in reducing noise. The average MSE value was approximately 32,11%, lower than that of the second-best method, AGGD. Regarding the PSNR, the proposed method also achieved the best results, with an average value of approximately 33.16 dB, superior to that of the other techniques. Regarding the SSIM, the average value across all noise levels was approximately 0.392. This value is considerably higher than those obtained using the different methods, further evidencing the superiority of the proposed approach in reducing noise.

Table 2 presents the performance results concerning the MSE, PSNR, and SSIM for MRI Brain Images 3 and 4 using different noise reduction methods based on adaptive wavelet thresholds. The data indicate that the proposed method outperforms all competing techniques across the metrics evaluated. In general, a combination of the linear prediction factor with the wavelet decomposition scale, as proposed in this study, proved to be more efficient than alternative techniques, especially at higher noise levels.

**Table 2:** MSE, PSNR(dB), and SSIM results for all wavelet methods; MRI Brain Images 3 and 4.

**Image**	***MRI Brain Image 3***					***MRI Brain Image 4***				
Noise variance (σ)	0.01	0.02	0.03	0.04	0.05	0.01	0.02	0.03	0.04	0.05
**MSE**										
*Proposed*	**25**	**30**	**30**	**31**	**31**	**26**	**30**	**32**	**34**	**35**
*VisuShrink*	219	375	528	676	826	220	380	535	685	829
*BayesShrink*	88	115	129	138	145	85	113	126	136	142
*Gaussian Kernel*	88	115	127	136	143	86	112	125	134	140
*Improved Threshold*	90	116	129	137	144	87	113	126	135	140
*AGGD*	30	34	36	37	38	31	37	39	40	42
**PSNR(dB)**										
*Proposed*	**34.09**	**33.43**	**33.30**	**33.21**	**33.16**	**34.05**	**33.21**	**33.03**	**32.83**	**32.69**
*VisuShrink*	24.72	22.39	20.90	19.83	18.96	24.71	22.34	20.85	19.78	18.95
*BayesShrink*	28.69	27.52	27.02	26.72	26.52	28.85	27.61	27.12	26.80	26.62
*Gaussian Kernel*	28.69	27.54	27.09	26.78	26.58	28.78	27.62	27.15	26.87	26.66
*Improved Threshold*	28.59	27.50	27.04	26.75	26.56	28.76	27.60	27.12	26.84	26.67
*AGGD*	33.29	32.76	32.56	32.47	32.37	33.18	32.50	32.19	32.06	31.94
**SSIM**										
*Proposed*	**0.486**	**0.409**	**0.367**	**0.333**	**0.313**	**0.478**	**0.397**	**0.348**	**0.313**	**0.289**
*VisuShrink*	0.430	0.346	0.300	0.271	0.247	0.412	0.319	0.272	0.241	0.220
*BayesShrink*	0.429	0.349	0.307	0.278	0.258	0.413	0.323	0.279	0.247	0.226
*Gaussian Kernel*	0.422	0.341	0.300	0.271	0.251	0.405	0.316	0.269	0.241	0.221
*Improved Threshold*	0.419	0.340	0.300	0.272	0.249	0.405	0.314	0.269	0.241	0.220
AGGD	0.395	0.319	0.279	0.253	0.232	0.375	0.290	0.247	0.220	0.202

The values in bold show better results compared to the other methods.

According to the values presented in Table 2, for MRI Brain Image 3, the average MSE obtained was 29 for the technique proposed, which is approximately 17% lower than that achieved with the AGGD method, the second-best performing technique. The proposed method also recorded an average PSNR of 33.44 dB and an SSIM of 0.382, the highest among the methods tested.

For MRI Brain Image 4, the proposed method achieved an average MSE of 31, reflecting an 18% reduction compared to that of the second-best method, AGGD. Regarding the PSNR, the average value was 33.16 dB, showing an improvement of 0.80 dB over the second-best result. SSIM averaged 0.365 with noise variations between 0.01 and 0.05, representing an improvement of 0.100 over the value of the AGGD method, the worst performer in this case. These results demonstrate the effectiveness of the proposed technique in reducing noise across different images and noise levels.

The results for the MSE, PSNR, and SSIM techniques for MRI Brain Images 5 and 6 are presented in Table 3. For MRI Brain Image 5, the proposed method achieved an average MSE of approximately 30,19%, lower than that obtained with the second-best method, AGGD. Concerning the PSNR, the proposed method recorded an average value of about 33.40 dB, an improvement of around 1 dB over that of AGGD. Regarding the SSIM, the proposed technique also yielded the best result, with an average value of 0.365, which represents an improvement of 0.102 compared to that obtained in the worst case, the AGGD method. For MRI Brain Image 6, the proposed method achieved an average MSE of 33,25%, lower than that of AGGD, the second-best technique. About the PSNR metric, the average value for the proposed method was approximately 32.90 dB, an improvement of around 1.20 dB over the value achieved with AGGD. For SSIM, the proposed method recorded an average of 0.442, outperforming the AGGD method by approximately 0.100.

**Table 3:** MSE, PSNR(dB), and SSIM results for all wavelet methods; MRI Brain Images 5 and 6.

**Image**	***MRI Brain Image 5***					***MRI Brain Image 6***				
Noise variance (σ)	0.01	0.02	0.03	0.04	0.05	0.01	0.02	0.03	0.04	0.05
**MSE**										
*Proposed*	**25**	**29**	**31**	**32**	**33**	**31**	**33**	**34**	**34**	**35**
*VisuShrink*	216	373	525	681	812	239	390	536	680	818
*BayesShrink*	85	112	126	136	141	82	107	120	130	137
*Gaussian Kernel*	85	112	125	133	140	82	106	119	129	134
*Improved Threshold*	87	112	126	134	141	82	106	120	128	134
*AGGD*	31	36	38	40	41	38	43	45	46	47
**PSNR(dB)**										
*Proposed*	**34.11**	**33.45**	**33.24**	**33.06**	**32.89**	**33.24**	**32.92**	**32.85**	**32.77**	**32.69**
*VisuShrink*	24.80	22.42	20.93	19.80	19.03	24.34	22.22	20.84	19.81	19.00
*BayesShrink*	28.85	27.63	27.12	26.80	26.63	29.00	27.85	27.33	27.00	26.78
*Gaussian Kernel*	28.83	27.64	27.16	26.88	26.67	28.98	27.86	27.37	27.04	26.84
*Improved Threshold*	28.72	27.63	27.13	26.86	26.63	29.01	27.87	27.34	27.06	26.85
*AGGD*	33.18	32.53	32.30	32.11	32.02	32.32	31.77	31.56	31.48	31.37
**SSIM**										
*Proposed*	**0.479**	**0.394**	**0.346**	**0.315**	**0.289**	**0.559**	**0.477**	**0.427**	**0.388**	**0.360**
*VisuShrink*	0.411	0.317	0.267	0.234	0.216	0.498	0.407	0.350	0.311	0.283
*BayesShrink*	0.413	0.321	0.276	0.245	0.223	0.515	0.423	0.366	0.330	0.301
*Gaussian Kernel*	0.405	0.311	0.266	0.237	0.216	0.509	0.414	0.358	0.319	0.292
*Improved Threshold*	0.403	0.313	0.266	0.239	0.216	0.509	0.413	0.358	0.320	0.292
AGGD	0.374	0.286	0.245	0.217	0.195	0.480	0.384	0.330	0.293	0.265

The values in bold show better results compared to the other methods.

### 3.2 Qualitative assessment

**Figure 4:**
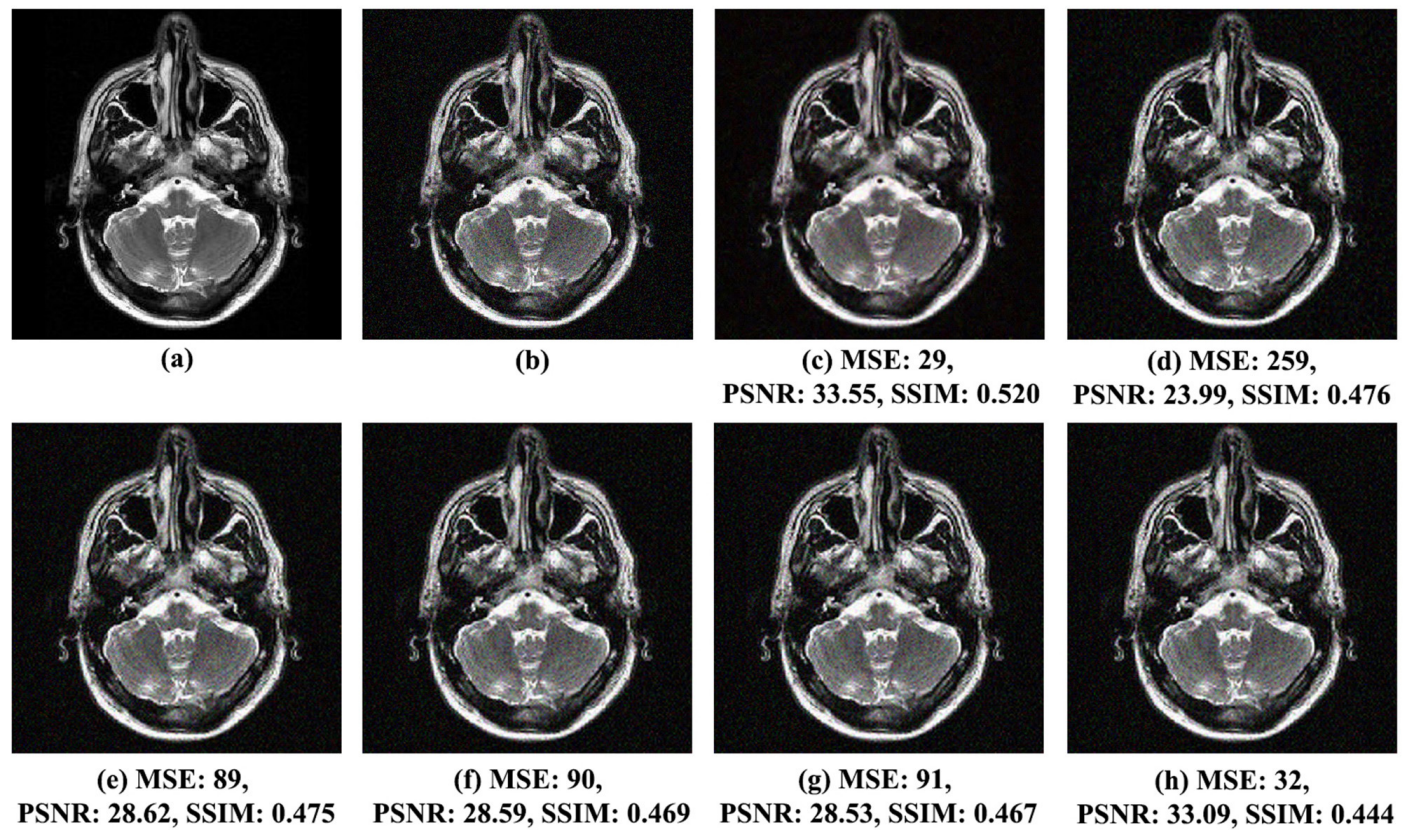
Comparison of noise reduction performance for MRI Brain Image 1 with noise variance of 0.01: **(A)** original image, **(B)** noisy image, **(C)** proposed method, **(D)** VisuShrink, **(E)** BayesShrink, **(F)** Gaussian Kernel, **(G)** improved threshold, and **(H)** AGGD.

**Figure 5:**
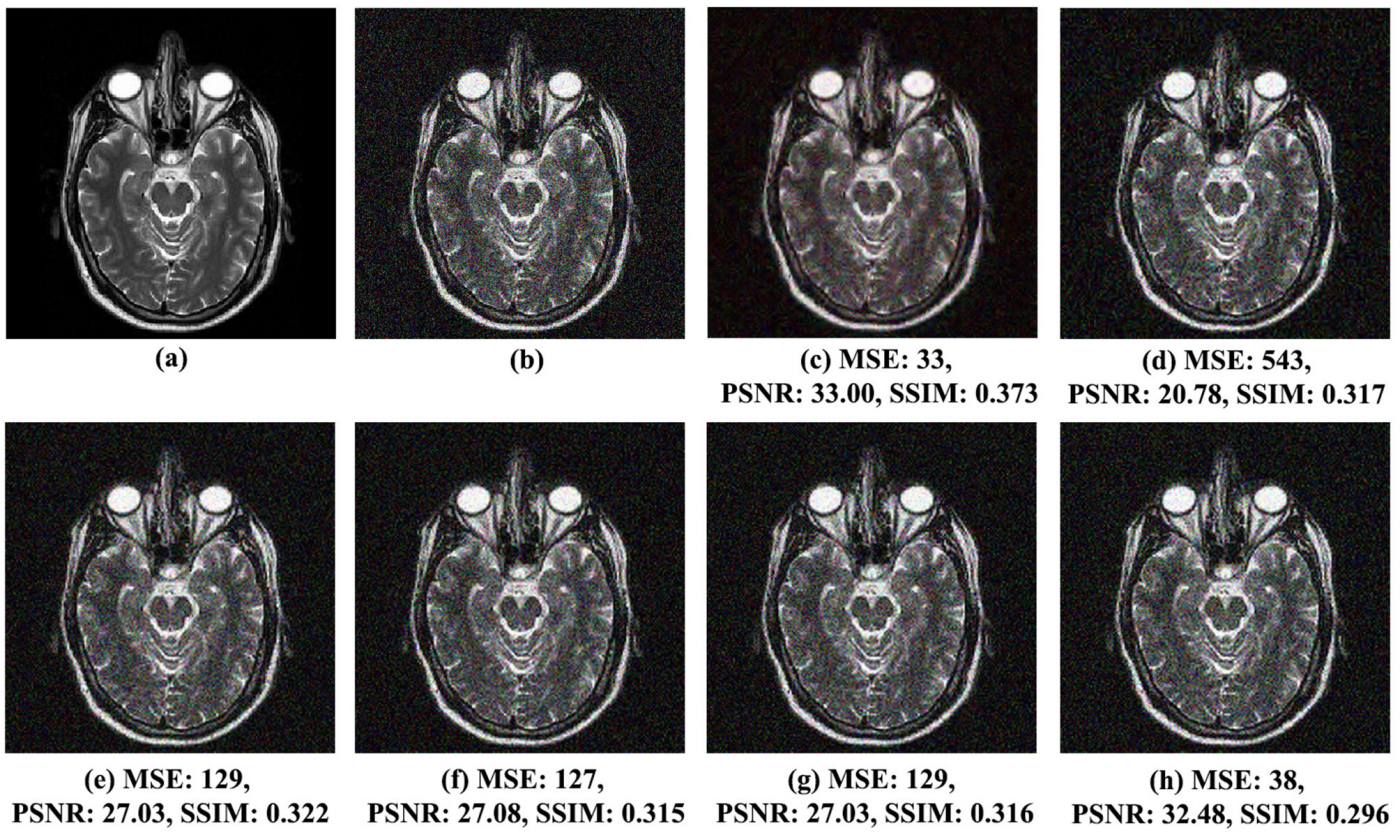
Comparison of noise reduction performance for MRI Brain Image 2 with noise variance of 0.03: **(A)** original image, **(B)** noisy image, **(C)** proposed method, **(D)** VisuShrink, **(E)** BayesShrink, **(F)** Gaussian Kernel, **(G)** improved threshold, and **(H)** AGGD.

**Figure 6:**
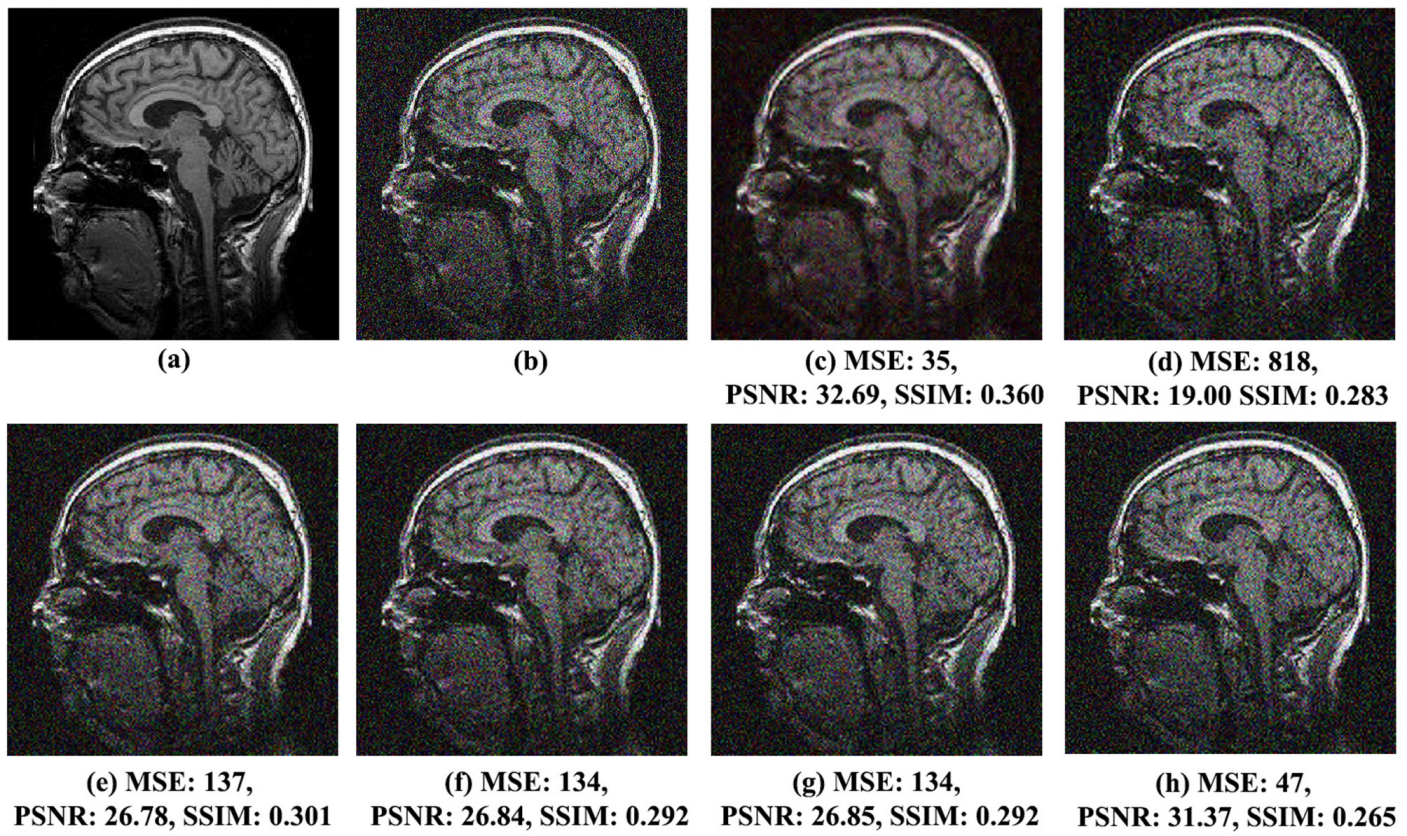
Comparison of noise reduction performance for MRI Brain Image 6 with noise variance of 0.05: **(A)** original image, **(B)** noisy image, **(C)** proposed method, **(D)** VisuShrink, **(E)** BayesShrink, **(F)** Gaussian Kernel, **(G)** improved threshold, and **(H)** AGGD.

The qualitative assessment of visual effects for the proposed noise reduction method and the other analyzed methods, *VisuShrink* (Donoho and Johnstone, 1994), *BayesShrink* (Chang et al., 2000), Gaussian Kernel (Sun et al., 2019), Improved Threshold (Zhang et al., 2019), and AGGD (Golilarz et al., 2020), comparing the original and noisy images, is shown in Figure 4 for MRI Brain Image 1 with Gaussian noise variance of 0.01, Figure 5 for MRI Brain Image 2 with Gaussian noise variance of 0.03, and Figure 6 for MRI Brain Image 6 with Gaussian white noise variance of 0.05.

Figures 4C–H present the qualitative results of the processed images using the wavelet transform with the biorthogonal wavelet base *bior3.9*. The proposed method, based on the linear prediction factor β, exhibits superior visual quality compared to the other methods. This is supported by the SSIM score of 0.520, which reflects excellent visual quality and is significantly higher than that achieved by the another techniques. In contrast, the AGGD method, which yielded the lowest SSIM score of 0.444, falls approximately 15%, inferior to the proposed method. The proposed approach effectively reduces noise in the reconstructed image, which is also evident in the MSE and PSNR metrics. The proposed method achieved an MSE of 29, the lowest across all methods. Furthermore, its PSNR was 33.55 dB, the best result compared to those of the other techniques. Therefore, the proposed method better preserves the structure and details of the processed image while reducing noise.

Similar observations were made for MRI Brain Images 2 and 6, illustrated in Figures 5C–H, 6C–H, respectively. In Figure 5C, MRI Brain Image 2, the proposed method again demonstrates superior visual quality, achieving an SSIM score of 0.373, approximately 21% higher than that of the AGGD method, which presented the lowest SSIM value of 0.296. For MRI Brain Image 6, as shown in Figure 6C, the proposed method achieved an SSIM value of 0.360, approximately 26% higher than that of AGGD, which yielded an SSIM of 0.265.

To compare the computational efficiency of the analyzed methods, we measured the processing time for each technique on a brain MRI image with a noise variance of 0.01. All noise reduction methods exhibited similar processing times: The proposed method was 0.72 seconds, the VisuShrink was 0.71 seconds, the BayesShrink was 0.71 seconds, the Gaussian Kernel was 0.78 seconds, the Improved Threshold was 0.85 seconds, and the AGGD was 0.75 seconds. While the processing times are comparable, the proposed method stands out due to its use of a linear prediction factor that analyzes both the original and noise images. This approach yields superior results in objective metrics and quantitative, and qualitative assessment.

The results presented demonstrate the strong performance of the proposed method for noise reduction in magnetic resonance brain images (Figure 3), even with variations in white Gaussian noise. The visual quality of the processed images, combined with the SSIM metric, highlights the method's ability to enhance texture, borders, and smooth regions, significantly outperforming the other evaluated methods.

## 4 Discussion and conclusion

In this paper, a noise reduction method for magnetic resonance brain imaging based on a linear prediction factor was developed to estimate the wavelet transform coefficients. Both quantitative and qualitative evaluations demonstrated the importance of using wavelet transforms in this context. The study provided an in-depth analysis of wavelet theory and its application in image noise reduction, focusing on adaptive wavelet thresholding techniques with a biorthogonal wavelet base. It emphasized the significance of these methods in reducing noise in magnetic resonance brain imaging and highlighted how this technology can significantly benefit medical applications by yielding conclusive results. By enhancing image clarity, the technique promotes better detection and more detailed visualization, which are essential for accurate diagnoses. The advanced exploration of wavelet transforms underscores their value as a fundamental tool for signal and image compression, as well as data processing, offering a more precise analysis of noise decomposition in images and facilitating improved visualization.

The proposed technique, which utilizes the linear prediction factor β, demonstrated superior performance across various metrics, achieving notable results in terms of averages and decomposition levels. This was particularly evident in the improved visual quality of the images, as reflected in the SSIM results. The proposed technique yielded the best outcomes for the biorthogonal wavelet base *bior3.9*, as highlighted by its top performance in SSIM, MSE, and PSNR evaluations. The proposed method enhances visual perception across all evaluation techniques, as evidenced in the experimental results, which show notable improvements in edge regions, smooth areas, and image texture.

For future work, there is potential to enhance the technique further by integrating adaptive filters, bilateral filtration, joint bilateral filtration, and the Wiener filter. These filters, especially adaptive filtering, integrated into the proposed method will make it possible to perform non-linear filtering that preserves image edges while smoothing noise, as they combine spatial and intensity similarity information, as well as the Wiener filter, which presents filtering based on decrease the mean squared error between the original and noise image, as it is inspired by on a statistical model of the original and noise signal in terms of mean, variance, and autocorrelation.

## Data Availability

The original contributions presented in the study are included in the article/supplementary material, further inquiries can be directed to the corresponding author.
